# Standardized Diagnostic Workup and Patient-Centered Decision Making for Surgery and Neck Dissection Followed by Risk-Factor Adapted Adjuvant Therapy Improve Loco-Regional Control in Local Advanced Oral Squamous Cell Carcinoma

**DOI:** 10.3389/fonc.2021.737080

**Published:** 2021-11-10

**Authors:** Gunnar Wichmann, Mykola Pavlychenko, Maria Willner, Dirk Halama, Thomas Kuhnt, Regine Kluge, Tanja Gradistanac, Sandra Fest, Theresa Wald, Bernd Lethaus, Andreas Dietz, Susanne Wiegand, Veit Zebralla

**Affiliations:** ^1^ Department of Otorhinolaryngology, Head and Neck Surgery, University Hospital Leipzig, Leipzig, Germany; ^2^ Department of Maxillofacial Surgery, University Hospital Leipzig, Leipzig, Germany; ^3^ Department of Radiation Oncology, University Hospital Leipzig, Leipzig, Germany; ^4^ Department of Nuclear Medicine, University Hospital Leipzig, Leipzig, Germany; ^5^ Department of Pathology, University Hospital Leipzig, Leipzig, Germany

**Keywords:** oral squamous cell carcinoma (OSCC), head and neck cancer, outcome research, elective neck dissection (ND), local control (LC), distant metastasis free survival (DMFS), overall survival (OS), multidisciplinary tumor board (MDTB)

## Abstract

**Background:**

Standardized staging procedures and presentation of oral squamous cell carcinoma (OSCC) patients in multidisciplinary tumor boards (MDTB) before treatment and utilization of elective neck dissection (ND) are expected to improve the outcome, especially in local advanced LAOSCC (UICC stages III–IVB). As standardized diagnostics but also increased heterogeneity in treatment applied so far have not been demonstrated to improve outcome in LAOSCC, a retrospective study was initiated.

**Methods:**

As MDTB was introduced into clinical routine in 2007, 316 LAOSCC patients treated during 1991-2017 in our hospital were stratified into cohort 1 treated before (*n*=104) and cohort 2 since 2007 (*n*=212). Clinical characteristics, diagnostic procedures and treatment modality of patients were compared using Chi-square tests and outcome analyzed applying Kaplan-Meier plots and log-rank tests as well as Cox proportional hazard regression. Propensity scores (PS) were used to elucidate predictors for impaired distant metastasis-free survival (DMFS) in PS-matched patients.

**Results:**

Most patient characteristics and treatment modalities applied showed insignificant alteration. Surgical treatment included significantly more often resection of the primary tumor plus neck dissection, tracheostomy and percutaneous endoscopic gastrostomy tube use. Cisplatin-based chemo-radiotherapy was the most frequent. Only insignificant improved disease- (DFS), progression- (PFS) and event-free (EFS) as well as tumor-specific (TSS) and overall survival (OS) were found after 2006 as local (LC) and loco-regional control (LRC) were significantly improved but DMFS significantly impaired. Cox regression applied to PS-matched patients elucidated N3, belonging to cohort 2 and cisplatin-based chemo-radiotherapy as independent predictors for shortened DMFS. The along chemo-radiotherapy increased dexamethasone use in cohort 2 correlates with increased DM.

**Conclusions:**

Despite standardized diagnostic procedures, decision-making considering clear indications and improved therapy algorithms leading to improved LC and LRC, shortened DMFS hypothetically linked to increased dexamethasone use had a detrimental effect on TSS and OS.

## Introduction

Surgery followed by postoperative radio- (Op+PORT) or platinum-based concomitant radio-chemotherapy (Op+PORCT) represent the recommended standard of care in local and/or loco-regional advanced oral squamous cell carcinoma (LAOSCC) in Germany. Definitive radiotherapy (RT) and concomitant radio-chemotherapy (CRT) are only recommended to LAOSCC patients diagnosed with very advanced disease without a chance to achieve by resection both aims, disease-free resection margins (R0) and good functional outcome. The general use of computed tomography (CT) imaging, in selected cases combined with positron-emission tomography (PET-CT) (1) together with standardized staging procedures (2) and presentation of LAOSCC patients in multidisciplinary tumor boards (MDTB) before treatment (3, 4) as well as utilization of elective neck dissection (ND) even in absence of suspect neck nodes (radiologic N0 category) are shown to improve outcome (5). The implementation of evidence-based decision-making for particular diagnostic and therapy according to institutional guidelines by adhering to NCCN (6) and ASCO guidelines (7) and the discussion of the individual case in the light of results obtained with the modern diagnostic and therapeutic procedures should improve survival rates especially in LAOSCC, as we recently demonstrated improved outcome since 2007 for neck squamous cell carcinoma of unknown primary (8). However, the now more patient-centered decision-making processes that consider individual preferences of the patient as well as more intense counselling of the patient that includes offering to get second opinion from another health care provider, sometimes associated with a delay in starting the treatment, and other factors may lead to increased heterogeneity in treatment applied and the individual clinical course of the patient and hence also influence the outcome. Our aim was to assess outcome differences before and after introduction of standardized diagnostic workup and patient-centered decision making for surgery and neck dissection followed by risk-factor adapted adjuvant therapy that was simultaneously implemented by establishing our MDTB.

## Materials and Methods

### Patients and Pathologic Tumor Data

**Figure 1:**
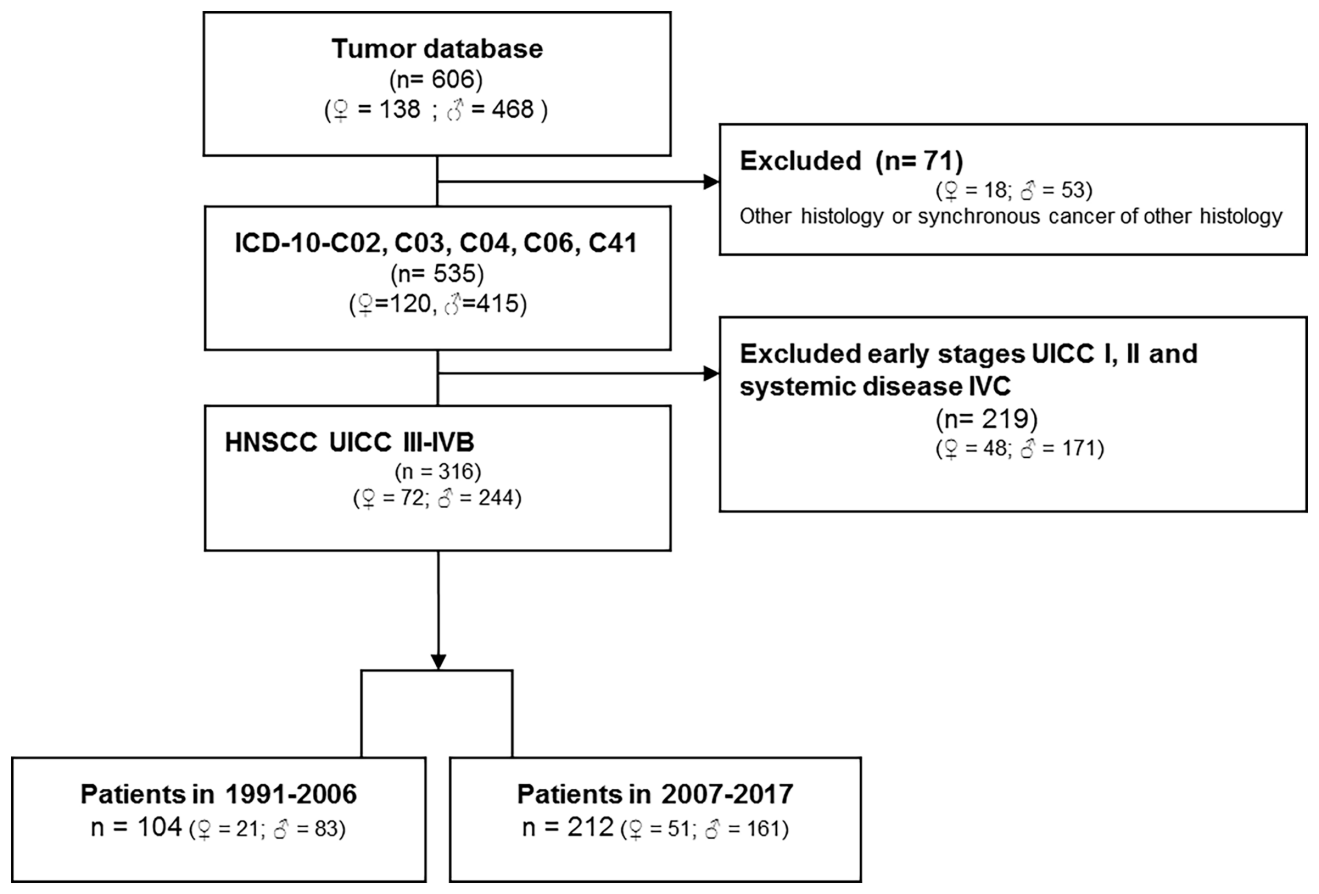
CONSORT diagram showing the selection of patients under study.

The tumor database of the ENT department of University Leipzig comprises data of 5,586 patients diagnosed with malignant disease. **Figure 1** (CONSORT diagram) summarizes eligibility criteria and the selection process. Eligibility criteria included: i) oral cancer as primary tumor site (ICD-10-C02, C03, C04, C06, C41); ii) patho-histological confirmed squamous cell carcinoma of advanced stage (UICC III-IVB according to TNM 2010; T1-T4N+ and T3-T4N0 (2)) excluding patients with distant metastasis (M1; UICC IVC); iii) absence of any prior or synchronous malignancy of other histology than SCC; iv) date of first diagnosis between 1991 and 2017; v) patho-histological report with information about the number of positive neck nodes (N+) and the N category. Patho-histological characteristics including ECE (+/-) and epidemiological risk factors (alcohol and tobacco smoking history) were recorded. The study was approved by the ethics committee of the University Leipzig the (votes 201-10-12072010 and 202-10-12072010), and conducted according to the guidelines of the Declaration of Helsinki. All patients provided written informed consent.

### Clinical Work-Up for LAOSCC

Clinical work-up for LAOSCC-P until 2006 (cohort 1) varied and included *e.g.* clinical examination, ultrasound sonography and other variable procedures (**Table 1**) before treatment. Since 2007 (cohort 2) clinical work-up was standardized and included, as recommended (6), clinical examination, ultrasound sonography, contrast-enhanced CT or even PET-CT/PET-MRI followed by panendoscopy including excision biopsies from suspect tissue.

**Table 1:** Baseline characteristics of the study population.

Characteristics		Total			Cohort 1			Cohort 2		*p* value†
		(*N*=316)			(*N*=104)			(*N*=212)		(*N*=316)
		*n* (%)			*n* (%)			*n* (%)		*n* (%)
Age (years)	<=50	78		(24.7)	28		(26.9)	50	(23.6)	0.5919
	<=60	112		(35.4)	38		(36.5)	74	(34.9)	
	<=70	72		(22.8)	23		(22.1)	49	(23.1)	
	<=80	44		(13.9)	14		(13.5)	30	(14.2)	
	>80	10		(3.2)	1		(1.0)	9	(4.2)	
Sex	Female	72		(22.8)	21		(20.2)	51	(24.1)	0.4415
	Male	244		(77.2)	83		(79.8)	161	(75.9)	
Tumor localization, stage, T & N category										
	Tongue (C02)	152		(48.1)	59		(56.7)	93	(43.9)	0.0145
	Mandible (C03)	27		(8.5)	2		(1.9)	25	(11.8)	
	Floor of mouth (C04)	116		(36.7)	36		(34.6)	80	(37.7)	
	Other (C06, C41)	21		(6.6)	7		(6.7)	14	(6.6)	
ICD-10 C02 *vs*	C02	152		(48.1)	59		(56.7)	93	(43.9)	0.0315
other	Other	164		(51.9)	45		(43.3)	119	(56.1)	
TNM 7^th^ ed. 2010^‡^,	Stage III	73		(23.1)	35		(33.7)	38	(17.9)	0.0009
UICC	Stage IVA	225		(71.2)	60		(57.7)	165	(77.8)	
	Stage IVB	18		(5.7)	9		(8.7)	9	(4.2)	
TNM 8^th^ ed. 2017^‡‡^,	Stage III	69		(21.8)	35		(33.7)	34	(16.0)	0.0001
UICC	Stage IVA	186		(58.9)	59		(56.7)	127	(59.9)	
	Stage IVB	61		(19.3)	10		(9.6)	51	(24.1)	
T categories^‡¦^										
TNM 7^th^ ed. 2010	T1	35		(11.1)	10		(9.6)	25	(11.8)	1.9×10^-5^
	T2	60		(19.0)	24		(23.1)	36	(17.0)	
	T3	65		(20.6)	33		(31.7)	32	(15.1)	
	T4a	148		(46.8)	31		(29.8)	117	(55.2)	
	T4b	8		(2.5)	6		(5.8)	2	(0.9)	
N categories TNM 7^th^ ed. 2010^‡^	N0	57		(18.0)	19		(18.3)	38	(17.9)	0.3642
	N1	70		(22.2)	28		(26.9)	42	(19.8)	
	N2	175		(55.3)	51		(49.0)	124	(58.5)	
	*N2a*	*9*		*(2.8)*	*2*		*(1.9)*	*7*	*(3.3)*	
	*N2b*	*91*		*(28.8)*	*31*		*(29.8)*	*60*	*(28.3)*	
	*N2c*	*75*		*(23.7)*	*18*		*(17.3)*	*57*	*(26.9)*	
	N3	14		(4.4)	6		(5.8)	8	(3.8)	
N categories TNM 8^th^ ed. 2017^‡‡^	N0	57		(18.0)	19		(18.3)	38	(17.9)	2.4×10^-5^
	N1	64		(20.3)	28		(26.9)	36	(17.0)	
	N2	138		(43.7)	50		(48.0)	88	(41.5)	
	*N2a*	12		(3.8)	2		(1.9)	10	(4.7)	
	*N2b*	74		(23.4)	31		(29.8)	43	(20.3)	
	*N2c*	52		(16.5)	17		(16.3)	35	(16.5)	
	N3a	10		(3.2)	6		(5.8)	4	(1.9)	
	N3b	47		(14.9)	1		(1.0)	46	(21.7)	
Grading	G1 and G2	233		(73.7)	76		(73.1)	157	(74.1)	0.3865
	G3 and G4	70		(22.2)	19		(18.3)	51	(24.1)	
	*Missing*	*13*		*(4.1)*	*9*		*(8.7)*	*4*	*(1.9)*	
R status	R0	184		(58.2)	36		(34.6)	148	(69.8)	0.0011
	R1	8		(2.5)	5		(4.8)	3	(1.4)	
	R2	1		(0.3)	1		(1.0)	-	(0)	
	no Op	92		(29.1)	32		(30.8)	60	(28.3)	
	*Missing*	*31*		*(9.8)*	*30*		*(28.8)*	*1*	*(0.5)*	
Pn status	Pn0	144		(45.6)	42		(40.4)	102	(48.1)	1.7×10^-4^
	Pn1	52		(16.5)	2		(1.9)	50	(23.6)	
	*Missing*	*120*		*(38.0)*	*60*		*(57.7)*	*60*	*(28.3)*	
L status	L0	72		(22.8)	34		(32.7)	38	(17.9)	3.8×10^-9^
	L1	132		(41.8)	14		(13.5)	118	(55.7)	
	*Missing*	*112*		*(35.4)*	*56*		*(53.8)*	*56*	*(26.4)*	
V status	V0	159		(50.3)	43		(41.3)	116	(54.7)	0.0232
	V1	40		(12.7)	4		(3.8)	36	(17.0)	
	*Missing*	*117*		*(37.0)*	*57*		*(54.8)*	*60*	*(28.3)*	
Any soft risk factor	None	59		(18.7)	33		(31.7)	26	(12.3)	3.4×10^-12^
	Any (Pn1, V1, L1)	145		(45.9)	15		(14.4)	130	(61.3)	
	*Missing*	*112*		*(35.4)*	*56*		*(53.8)*	*56*	*26.4)*	
ECE^§^ with N0	ECE-	66		(20.9)	5		(4.8)	61	(28.8)	2.1×10^-6^
	ECE+	53		(16.8)	1		(1.0)	52	(24.5)	
	No ECE (N0)	57		(18.0)	19		(18.3)	38	(17.9)	
	*Missing*	*140*		*(44.3)*	*79*		*(76.0)*	*61*	*(28.8)*	
p16 Status	p16-	105		(33.2)	2		(1.9)	103	(48.6)	0.2691
	p16+	15		(4.7)	1		(1.0)	14	(6.6)	
	*Missing*	*196*		*(62.0)*	*101*		*(97.1)*	*95*	*(44.8)*	
Smoking	Never	37		(11.7)	7		(6.7)	30	(14.2)	0.2776
	Former	32		(10.1)	6		(5.8)	26	(12.3)	
	Current	217		(68.7)	62		(59.6)	155	(73.1)	
	*Missing*	*30*		*(9.5)*	*29*		*(27.9)*	*1*	*(0.5)*	
Smoking categories	<5PY	42		(13.3)	8		(7.7)	34	(16.0)	0.5729
	<15PY	18		(5.7)	6		(5.8)	12	(5.7)	
	<35PY	112		(35.4)	29		(27.9)	83	(39.2)	
	<45PY	62		(19.6)	18		(17.3)	44	(20.8)	
	<55PY	32		(10.1)	10		(9.6)	22	(10.4)	
	>=55PY	16		(5.1)	2		(1.9)	14	(6.6)	
	*Missing*	*34*		*(10.8)*	*31*		*(29.8)*	*3*	*(1.4)*	
Smoking quintiles	<=10PY	53		(16.8)	11		(10.6)	42	(19.8)	0.7786
	<=25PY	63		(19.9)	15		(14.4)	48	(22.6)	
	<=32PY	51		(16.1)	13		(12.5)	38	(17.9)	
	<=40PY	62		(19.6)	19		(18.3)	43	(20.3)	
	>40PY	53		(16.8)	15		(14.4)	38	(17.9)	
	*Missing*	*34*		*(10.8)*	*31*		*(29.8)*	*3*	*(1.4)*	
Alcohol drinking	Never	27		(8.5)	8		(7.7)	19	(9.0)	0.8919
	Former	37		(11.7)	9		(8.7)	28	(13.2)	
	Current	222		(70.3)	60		(57.7)	162	(76.4)	
	*Missing*	*30*		*(9.5)*	*27*		*(26.0)*	*3*	*(1.4)*	
Alcohol categories	0 g/day	27		(8.5)	8		(7.7)	19	(9.0)	0.0001
	1-30 g/day	65		(20.6)	5		(4.8)	60	(28.3)	
	31-60 g/day	47		(14.9)	9		(8.7)	38	(17.9)	
	>60 g/day	146		(46.2)	54		(51.9)	92	(43.4)	
	*Missing*	*31*		*(9.8)*	*28*		*(26.9)*	*3*	*(1.4)*	
Age at diagnosis	Mean (95% CI)	57.6		(55.5 - 59.7)			58.8	(57.1 - 60.4)		0.3751
Pack years	Mean (95% CI)	29.3		(25.7 - 32.8)			28.0	(25.6 - 30.4)		0.5694
N assessed	Mean (95% CI)	10.1		(6.9 - 13.4)			26.9	(23.9 - 29.9)		4.4×10^-12^
N+	Mean (95% CI)	1.1		(0.8 - 1.4)			2.0	(1.6 - 2.4)		0.0003

^†^ Pearson’s Chi-square (χ^2^) test for contingency tables; ^‡^ TNM staging according to 7^th^ ed. 2010 (2); ^‡‡^ TNM staging according to 8^th^ ed. 2017; ^‡¦^ T categories according to TNM 8^th^ edition now are considering depth of invasion not completely recorded in both cohorts; ^§^ ECE, extracapsular extension; heteroscedastic t-test for cardinal metric data. Distributions are shown with number of cases and percentage in brackets. Missing values in table are not included in analyses and therefore presented italic.

### Decision-Making Process in the MDTB

Our weekly MDTB established in 2007 comprises all professions involved in the diagnostics and therapy of head and neck cancer patients. These are head and neck surgeons from the departments of ENT and maxillofacial surgery, radiologists and a board-certified nuclear radiologist, pathologist, medical oncologist, hematologic oncologist, radiation oncologist, prosthodontic dentist, and specialists from other departments, whenever required. The decision-making process in the MDTB for treatment of pathologic confirmed LAOSCC followed NCCN and ASCO guidelines (6, 7) or participation in open clinical trials including randomized controlled trials (RCT). Briefly, radiologist and nuclear medicine specialist presented all radiological imaging. Since 2007 (cohort 2), the pre-therapeutic MDTB discussed results of diagnostic procedures. Whenever patients where eligible for a RCT, offering participation was consented. According to guidelines (6), the MDTB mostly recommended ND as part of the surgical treatment.

After ND, the pathologist defined ECE being present whenever a capsule was missing (soft tissue deposit) or a disrupted lymph node capsule was visible macroscopically or microscopically (9). Every initially according to TNM 7^th^ ed. 2010 staged patient was reclassified according to TNM 8^th^ ed. 2017 (**Table 1**).

Considering the pathologic report as well as general health and comorbidity of the patient, the post-surgical MDTB consented a recommendation for treatment according to NCCN-Guidelines (6, 10). Smaller LAOSCC without local metastases (N0) and clear margins (R0 >5 mm) were treated by surgery alone (Op). However, most LAOSCC due to local metastases (N+) and/or extension of the primary required adjuvant treatment and received post-operative (adjuvant) radiotherapy (Op+PORT) or radio-chemotherapy (Op+PORCT). However, definitive primary radiotherapy (pRT) or concomitant chemo-radiotherapy (CRT) were recommended, whenever R0 resection and good functional outcome seemed to be impossible to achieve or were performed according to the patient’s preference, whenever he denied extensive surgery and reconstruction.

Best supportive care +/- palliative treatment was offered to patients without curative treatment options or if refused by the patient.

### Treatment Modalities

The treatment modalities applied to LAOSCC patients of cohort 1 and 2 are shown in **Table 2**. Also since 2004 intensity-modulated radiotherapy (IMRT) was available and used for PORT and PORCT, pRT and CRT. Irradiation plans for pRT and CRT without upfront surgery were scheduled to achieve 70 to 72 Gy totally in the gross tumor volume given in 35 fractions within 7 weeks. Cisplatin-based CRT used 3 cycles of single cisplatin infusions (100 mg/m^2^ at days 1, 22, and 43). In cohort 2, the LAOSCC-P with ND and detection of only unilateral N+ (N2b) without risk factors present (up to 2 N+ <6 cm, no ECE, R0/no incision biopsy) received PORT of 60 Gy ipsilateral and 50 Gy contralateral, independent from ND also of the unaffected site or not. Irradiation after resection of a single node without risk factors (<6 cm, no ECE, R0/no incision biopsy) was unilateral 60 Gy (8, 9). Whenever risk factors for local recurrence (bilateral N+, *i.e.* N2c, or one node ≥6 cm, *i.e.* N3, or ECE+, R1) were detected, bilateral irradiation with 64 Gy was accompanied by concomitant cisplatin (8, 9). Cisplatin was given either in up to 3 cycles of single infusions (100 mg/m^2^ at days 1, 22, and 43) or fractionated in five daily doses of 20 mg/m^2^ (days 1-5, 22-26, and 43-47) (8, 9). To reduce acute toxicity and combat cisplatin-related side effects, the latter regimen was predominantly used since 2007 (**Table 2**). To prevent vomiting and unwanted side effects of CRT and PORCT, dexamethasone was given adjuvant before and during infusion.

**Table 2:** Treatment and outcome in advanced squamous cell carcinoma in cohort 1 (1993-2006) and 2 (2007-2017).

Characteristics		Total		Cohort 1		Cohort 2		*p* value†
		(*N*=316)		(*N*=104)		(*N*=212)		(*N*=316)
		*n* (%)		*n* (%)		*n* (%)		*n* (%)
Therapy concept (2 groups)	Curative	272	(86.1)	89	(85.6)	183	(86.3)	0.8575
	Palliative or incomplete	44	(13.9)	15	(14.4)	29	(13.7)	
Therapy concept (3 groups)	Curative	272	(86.1)	89	(85.6)	183	(86.3)	0.7940
	Palliative	32	(10.1)	10	(9.6)	22	(10.4)	
	Incomplete	12	(3.8)	5	(4.8)	7	(3.3)	
Tracheostomy	No	170	(53.8)	81	(77.9)	89	(42.0)	1.7×10^-9^
	Yes	146	(46.2)	23	(22.1)	123	(58.0)	
PEG	No	128	(40.5)	63	(60.6)	65	(30.7)	3.5×10^-7^
	Yes	188	(59.5)	41	(39.4)	147	(69.3)	
Neck dissection (yes or no)	No ND	104	(32.9)	44	(42.3)	60	(28.3)	0.0127
	SND, mRND, RND	212	(67.1)	60	(57.7)	152	(71.7)	
Neck dissection	No ND	104	(32.9)	44	(42.3)	60	(28.3)	0.0003
	SND	194	(61.4)	49	(47.1)	145	(68.4)	
	RND, mRND	18	(5.7)	11	(10.6)	7	(3.3)	
Neck dissection and Op	No Op and no ND	91	(28.8)	32	(30.8)	59	(27.8)	1.0×10^-5^
	Op or ND	13	(4.1)	12	(11.5)	1	(0.5)	
	Op and ND	212	(67.1)	60	(57.7)	152	(71.7)	
Op (yes or no) ‡	No Op	91	(28.8)	32	(30.8)	59	(27.8)	0.5877
	Op	225	(71.2)	72	(69.2)	153	(72.2)	
RT and RChT *vs* none	None	64	(20.3)	22	(21.2)	42	(19.8)	0.7802
	RT, RChT	252	(79.7)	82	(78.8)	170	(80.2)	
RT *vs*. RChT *vs*. none	None	64	(20.3)	22	(21.2)	42	(19.8)	0.7322
	RT	136	(43.0)	47	(45.2)	89	(42.0)	
	RChT	116	(36.7)	35	(33.7)	81	(38.2)	
Therapy modality (detail)	no RT	64	(20.3)	22	(21.2)	42	(19.8)	0.5320
	PORT	93	(29.4)	31	(29.8)	62	(29.2)	
	PORCT	75	(23.7)	23	(22.1)	52	(24.5)	
	RT	43	(13.6)	16	(15.4)	27	(12.7)	
	CRT	34	(10.8)	12	(11.5)	22	(10.4)	
	IC+Op+POR(C)T^‡^	7	(2.2)	0	(0)	7	(3.3)	
Chemotherapy	CRT Carboplatin	1	(0.9)	1	(2.9)	0	(0)	2.9×10^-5^
	CRT Cisplatin	28	(24.1)	8	(22.9)	20	(24.7)	
	RT Cetuximab	2	(1.7)	0	(0)	2	(2.5)	
	CRT other chemo	3	(2.6)	3	(8.6)	0	(0)	
	PORT Carboplatin	7	(6.0)	7	(20.0)	0	(0)	
	PORT Cisplatin	60	(51.7)	14	(40.0)	46	(56.8)	
	PORT Cetuximab	5	(4.3)	0	(0.0)	5	(6.2)	
	PORT other chemo	3	(2.6)	2	(5.7)	1	(1.2)	
	IC+Op+POR(C)T^‡^	7	(6.0)	0	(0)	7	(8.6)	
	*No chemotherapy*	*200*		*69*		*131*		
Overall survival	Alive	147	(46.5)	42	(40.4)	105	(49.5)	0.1257
	Dead	169	(53.5)	62	(59.6)	107	(50.5)	
Overall survival	Alive	147	(46.5)	42	(40.4)	105	(49.5)	0.2971
	NCRD	55	(17.4)	21	(20.2)	34	(16.0)	
	CRD	114	(36.1)	41	(39.4)	73	(34.4)	
Tumor-specific survival (TSS) **^§^**	Alive or NCRD	223	(70.6)	67	(64.4)	156	(73.6)	0.0931
	CRD	93	(29.4)	37	(35.6)	56	(26.4)	
Event-free survival (EFS)	No event	99	(31.3)	25	(24.0)	74	(34.9)	0.0503
	event	217	(68.7)	79	(76.0)	138	(65.1)	
Disease-free survival (DFS)	Disease-free	164	(51.9)	51	(49.0)	113	(53.3)	0.4759
	Relapse or CRD	152	(48.1)	53	(51.0)	99	(46.7)	
Progression-free survival (PFS)	None	162	(51.3)	51	(49.0)	111	(52.4)	0.5790
	Relapse or PD	154	(48.7)	53	(51.0)	101	(47.6)	
LC	None	201	(63.6)	57	(54.8)	144	(67.9)	0.0227
	Relapse, PD	115	(36.4)	47	(45.2)	68	(32.1)	
NC	None	247	(78.2)	80	(76.9)	167	(78.8)	0.7082
	Relapse, PD	69	(21.8)	24	(23.1)	45	(21.2)	
LRC	None	191	(60.4)	55	(52.9)	136	(64.2)	0.0542
	Relapse, PD	125	(39.6)	49	(47.1)	76	(35.8)	
DC	None	272	(86.1)	98	(94.2)	174	(82.1)	0.0033
	Relapse, PD	44	(13.9)	6	(5.8)	38	(17.9)	
Other cancer entity	None	306	(96.8)	102	(98.1)	204	(96.2)	0.3772
	Other cancer	10	(3.2)	2	(1.9)	8	(3.8)	
Time to intervention (d)	Mean (95% CI)	23.2	(19.5 - 26.9)		32.8 (29.3 - 36.4)			0.0002
Therapy interval (d)	Mean (95% CI)	56.4	(46.5 - 66.2)		59.9 (53.9 - 65.9)			0.5459

^†^ Pearson’s Chi-square (χ^2^) test for contingency tables; ^‡^ IC+Op+POR(C)T TPF-induction-chemotherapy followed by surgery and postoperative radiotherapy or radio-chemotherapy; Op, only surgery; Op+PORT, Op followed by postoperative radiotherapy; Op+PORCT, Op followed by postoperative radio-chemotherapy; RT, definitive radiotherapy alone or PORT; RChT, concurrent radio-chemotherapy or PORCT; CRT, concurrent radio-chemotherapy; other, 3 cycles 40 mg/m^2^ taxol or mitomycin ± 5-fluorouracil (not further specified); § OS, overall survival; TSS, tumor-specific survival; EFS, event-free survival; DFS, disease-free survival; PFS, progression-free survival; LC, local control; ¶ heteroscedastic t-test for cardinal metric data. Missing values in table are not included in analyses and therefore presented italic.

### Statistical Analysis and Propensity-Score Matching

Statistical analyses using SPSS version 24 (11) included *Pearson’s Chi*-square (*χ* ^2^) tests to assess differences between categorical variables. Time-dependent covariates were measured from date of diagnosis to date of event. They included overall survival (OS; the time span from diagnosis until death of any cause by censoring patients alive at end of follow-up), tumor-specific survival (TSS; the time span from diagnosis until cancer-related death censoring patients alive at end of follow-up or death from other cause) and event-free survival (EFS; the interval from date of diagnosis until relapse or death from any cause, censoring patients at time of last follow-up alive without signs of any cancer). Disease-free survival (DFS) was measured from date of R0 resection or receipt of the last irradiation dose applied in PORT or PORCT in R1 resected cases or definitive pRT and CRT until the date of either relapse or cancer-related death censoring patients alive at last follow up without signs of disease.

Progression free survival (PFS) was defined as the time span from diagnosis until relapse or cancer-related death censoring patients alive at end of follow-up or death from other cause. Local control (LC) was measured as the time span from diagnosis until local recurrence or second primary squamous cell carcinoma in the head and neck region; nodal control (NC) as time to relapse in the draining neck nodes (N+ only). We measured loco-regional control (LRC) as the time from diagnosis until loco-regional relapse (sum of local and nodal relapse), and distant control (DC) as distant metastasis-free survival (DMFS), the time to diagnosis of distant metastasis (M1), censoring all other PFS events at time of last follow-up. Outcome differences between groups were analyzed using KM cumulative survival plots and log-rank tests. Univariate and multivariate Cox regression models were utilized to estimate a covariate’s hazard ratio (*HR*) and to identify independent predictors (*Pi*) of PFS, LC, LRC, DC, EFS, DFS, TSS, and OS. *P* values below 0.05 in 2-sided tests were considered being significant.

Logistic regression and propensity-score matching (PS-matching) was used to identify patients with identical or most similar characteristics, and 1:1 PS-matching was performed using SPSS version 24 (11) with a caliper width of 0.1 standard deviations of the linear predictor (12).

## Results

**Table 1** shows the characteristics of patients in cohort 1 (104 patients) and 2 (212 patients). Whereas most epidemiologic risk factors remained mostly unchanged, significant differences are found in the localization of the primary advanced OSCC with reduced frequency of tongue cancer and increased in the mandible, associated with increased frequency in T4 cancer (+25.4%), N2 categories (especially N2c, +9.6%), and UICC stage IVA (+20.1%). According to increased frequency of ECE+ in cohort 2 (*p*=2.1∙10^-6^) and by applying TNM 2017 (13), frequencies in N categories changed significantly (all *p*<0.0001): N1 and N2b were reduced (-9.9% and -9.5%), whereas the (new) category N3b increased by 20.7% having the highest impact on the also increased frequency in stage IVB (+14.5%). Overall, the information provided in pathology reports since 2007 was more comprehensive and in all cases treated by surgical resection included data about the number of analyzed neck nodes, resection margins as well as information about perineural (Pn1), lymphatic (L1) or vascular infiltration (V1) of the primary lesion or absence of those (Pn0, L0, and V0, respectively). In line with increased use of (according to institutional guidelines standardized) neck dissection, the number of neck nodes examined (mean and 95% confidence interval) increased from 10.1 (6.9-13.4) to 26.9 (23.9-29.9; *p*=4.4∙10^-12^) and led to increased numbers of N+ identified in cohort 2 (2.0, 95% CI 1.6-2.4) compared to cohort 1 (1.1, 95% CI 0.8-1.4; *p*=0.0003).

We achieved the goal of pre-therapeutic presentation of all head and neck cancer patients. Since 2007 more than 95% of all new diagnosed head and neck cancer patients were pre-therapeutic discussed in the MDTB and additionally post-therapy after availability of the pathology report for decision-making towards adjuvant (post-operative) treatment. Cisplatin-based PORCT was used more frequently. In addition, use of cetuximab added to pRT or PORT emerged as new treatment option for LAOSCC-P with rather poor organ function. Aiming on reducing chemo-related side effects, dexamethasone and histamine-receptor ± neurokinin inhibitors were used in general and treatment protocols modified to reduce acute toxicity. The higher fractionated schema applying the total cisplatin dose in 2 to 3 cycles of five daily doses each of 20 mg/m^2^ (days 1-5, 22-26, and 43-47) was more frequently used since 2007 (cohort 2).

The therapy concepts applied to cohorts 1 and 2 as well as treatment modalities, Op, Op+PORT or Op+PORCT, or treatment without surgery by pRT or CRT, almost differed not significantly in frequency but with some exceptions (**Table 2**). Related to standardized work-up and the adherence to standard operating procedures (SOP) and internal guidelines, the surgical treatment included significantly more often the use of neck dissections (ND) and in particular selective ND (SND) as well as surgical placement of tracheostomas and percutaneous endoscopic gastrostomy (PEG) feeding tubes (**Table 2**). Besides 7 patients treated in an induction-chemotherapy RCT with 3 cycles TPF (docetaxel, cisplatin, 5-fluorouracil) before surgical resection, the only significant changes observed in treatment modality frequencies were the reduced use of carboplatin and significant increase in cisplatin-based PORCT and also use of cetuximab added to PORT (**Table 2**). To reduce acute toxicity and combat cisplatin-related side effects, the latter regimen was predominantly used since 2007 (**Table 2**). In parallel, adjuvant treatment with dexamethasone was given during CRT and PORCT over a mean total time of 8.5 (95% CI 7.6 - 9.4) days in cohort 1 and 10.1 (95% CI 9.3 - 10.8) days in cohort 2 (*p*=0.0102). The mean total dexamethasone doses of patients receiving CRT and PORCT were 100.1 (95% CI 87.2 - 113.0) mg dexamethasone in cohort 1 and 119.6 (95% CI 110.5 - 128.7) mg dexamethasone in cohort 2 (*p*=0.0203).

**Figure 2:**
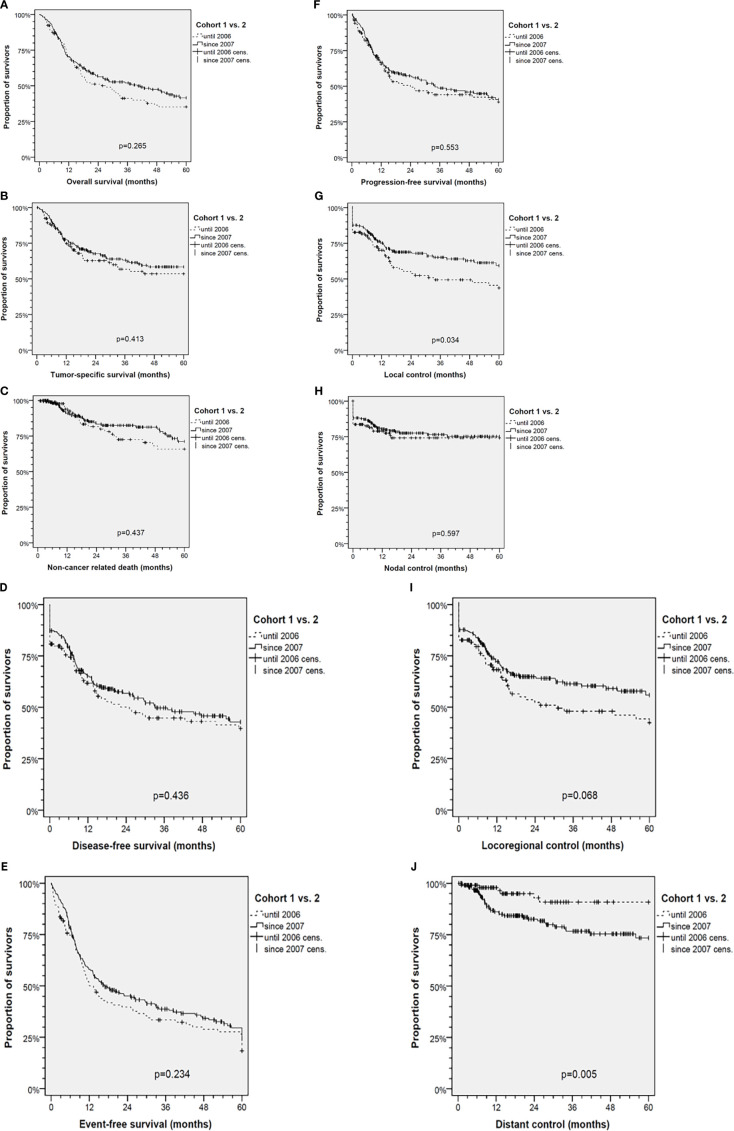
Kaplan-Meier cumulative survival analyses of advanced oral squamous cell carcinoma (OSCC) patients before (Cohort 1, 1993-2006) and after standardization of diagnostic workup and therapy (Cohort 2, 2007-2017) for **(A)** overall survival; **(B)** tumor-specific survival; **(C)** survival according to non-cancer death/death from other cause; **(D)** disease-free survival; **(E)** event-free survival; **(F)** progression-free survival; **(G)** local control; **(H)** nodal control; **(I)** loco-regional control; and **(J)** distant control. *P* values shown are from 2-sided log-rank tests.

Comparisons revealed increased numbers in T4 and higher N categories accompanied by impaired distant control (DC; *p*=0.0033) after 2006. The outcome, however, differed significantly regarding improved local (LC; *p*=0.0227) and loco-regional control (LRC; *p*=0.0542). Only insignificant improved DFS, PFS and EFS as well as slightly improved TSS and OS were detectable (**Figure 2**).

The diagnosis of distant metastasis (M1) in cohort 2 increased in parallel to standardized follow-up and higher frequency of post-therapeutic CT-imaging including chest and abdomen or even PET-CT imaging. Whereas in cohort 1 only 1 of 6 DC events (16.7%) was detected before loss in LRC, this was the case in 18 of 38 DC events (47.4%) in cohort 2 (*p*=0.158). In line with earlier detection and independent diagnosis of distant failure, the frequency of M1 not accompanied by loss in LC, NC or LRC was increased and the survival of patients after M1 diagnosis prolonged (mean 3.5, 95% CI 0.9 – 6.1, *vs*. 5.5, 95% CI 3.3 – 7.8 months in cohort 1 and 2; *p*=0.288).

**Figure 3:**
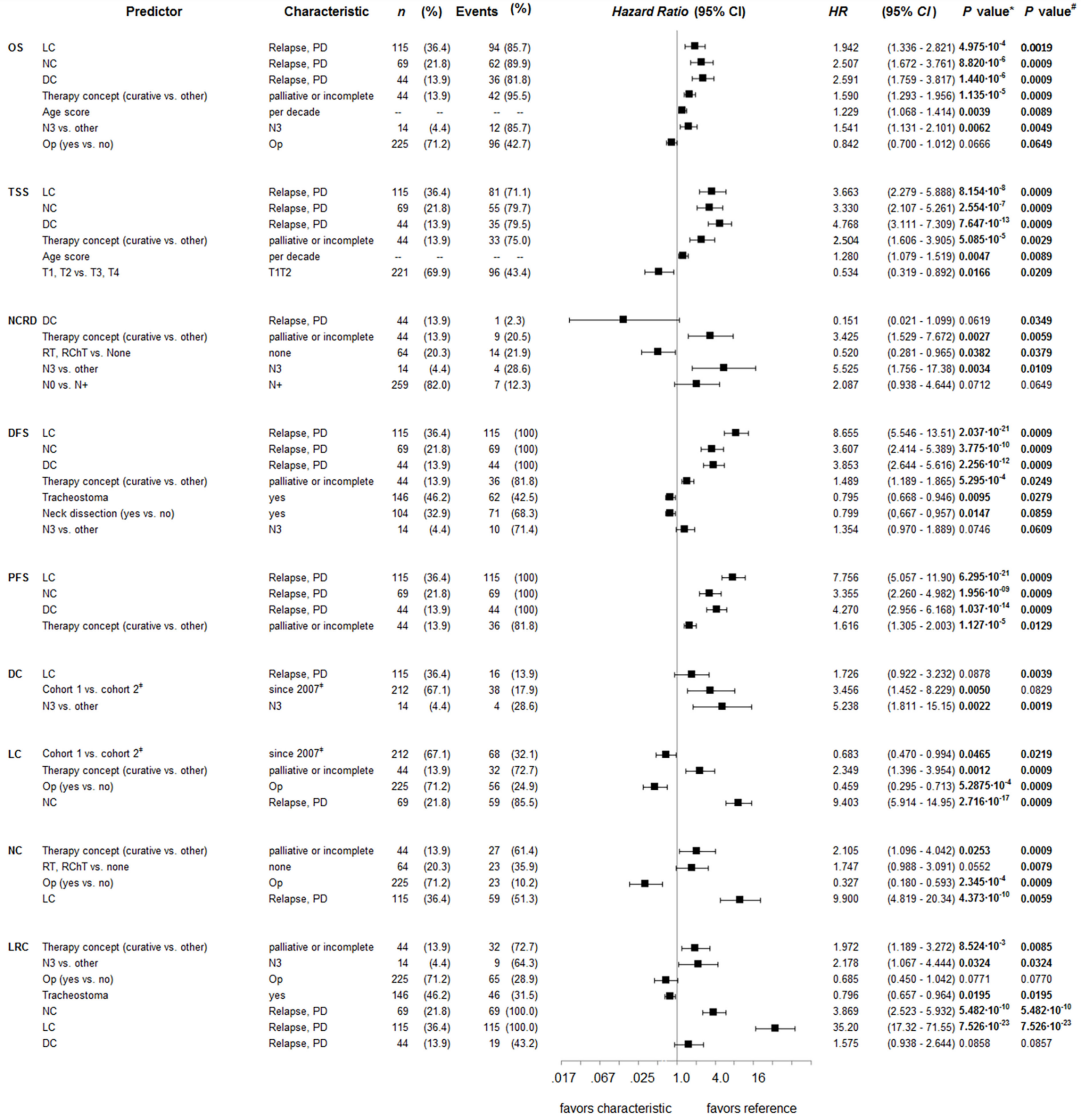
Forest plots for outcome predictors in advanced oral squamous cell carcinoma according to multivariate Cox proportional hazard regression analyses. Shown are hazard ratios (HR) and 95% confidence intervals for OS, overall survival; TSS, tumor-specific survival; NCRD, survival according to non-cancer death/death from other cause; DFS, disease-free survival; PFS, progression-free survival; DC, distant control; LC, local control; NC, nodal control; LRC, loco-regional control. *, *P* values for independent predictors in the Cox proportional hazard model of highest significance; ^#^, *P* values from bootstrap validation of the same Cox proportional hazard model applying 1,000 iterations. ;^#‡^Cohort 2 comprises patients diagnosed since 2007 (standardized workup and prediagnostic presentation in the multidisciplinary tumor board).

Kaplan-Meier plots of cumulative survival (**Figure 2**) with the only exception of DC show improved outcome of cohort 2. LC was significantly improved (*p*=0.034), but DC reduced (*p*=0.005). The net effect, however, was slightly (insignificant) improved DFS, PFS, EFS, TSS and OS. To clarify reasons for opposing trends respective to LC and DC, multivariate analyses applying Cox proportional hazard models were used (**Figure 3**). DFS besides being dependent on LC, NC and DC and per-protocol completed curative treatment was found being improved in patients who had ND and tracheostomy but impaired in those with N3. LC itself has a predictor in use of Op, belonging to cohort 2, and NC (nodal control); reciprocally, NC was dependent on LC and improved by applying Op and RT or CRT (independent from being used in postoperative or definitive setting). LC, despite the opposing trends in cohort 1 and 2, predicted DC. To solve the problem of improved LC in cohort 2 and despite improved LC reduced DC, analyses in propensity-score (PS) matched patients were done.

In the prior univariate analyses applying log-rank tests to Kaplan-Meier analyses or Cox proportional hazard models, we identified various predictive factors for DC/DMFS: high level of smoking (pack years), alcohol consumption, T1/T2 *vs.* T3/T4, N0 *vs.* N+, N3 *vs.* other, higher age at diagnosis, localization of the primary OSCC, male sex, alcohol consumption, and tobacco smoking. Therefore, a significantly higher prevalence of these factors in LAOSCC-P in cohort 2 had to be expected. A 1:1 PS-matching with a caliper width of 0.1 standard deviations of the linear predictor (12) was used to identify 70 LAOSCC-P in each cohort with according to *z*-scores nearly identical profile in the before mentioned characteristics. We applied multivariate Cox proportional hazard regression models for analysis of DC in these 140 cases (70 PS-matched patients from each of the two cohorts). We used the conditional stepwise-forward method in Cox proportional hazard regression to identify those covariates exerting strongest impact on DC in the PS-matched cases, the three covariates N3, cisplatin, and cohort 1 or 2. By including them, all other covariates (sex, age at diagnosis, level of daily alcohol consumption, history of tobacco smoking and pack years smoked, localization of the primary as well as individual treatment components [surgical operation, neck dissection, RT or CRT] applied or not as well as T and N categories, and also LC and NC) no longer had any impact on DC in these PS-matched patients. The exclusion of the formerly predictive covariates demonstrates absence of relevant residual confounding. The final model automatically built using the conditional stepwise-forward method extracted only 3 independent predictors for losing DC: belonging to cohort 2 (*HR* 4.25, 95% CI 1.41-12.85; *p*=0.0103); N3 (*HR* 6.23, 95% CI 1.66-23.44; *p*=0.0068); and cisplatin-based chemo-radiation (*HR* 1.96, 95% CI 0.78-4.94; *p*=0.1531). Bootstrapping utilizing 1,000 iterations revealed significance of the model (*p*=4.99∙10^-5^) and of these 3 independent predictors (all *p*<0.001) within the PS-matched LAOSCC-P. Therefore, being treated with cisplatin-based chemo-radiation is an independent predictor of DC loss but, however, not itself a significant contributor in our sample of LAOSCC-P.

## Discussion

Decision-making after standardized diagnostics in the MDTB can improve the efficiency of multidisciplinary patient management. Our MDTB allowed for implementation of clinical practice guidelines and quality control for diagnostic workflow, decision-making and therapy. Moreover, it helped to capture cases for clinical trials and allowed for quicker translation of their findings into our daily practice. These additional efforts should improve survival. Indeed, we found an improved outcome of LAOSCC patients diagnosed and treated in our university hospital after establishing the MDTB, and significantly improved LRC in particular. This confirms findings in our university hospital (8) and of other centers (14). After introduction of a MDTB at the University of Philadelphia the disease-specific survival of patients with head and neck cancer increased significantly from 52% to 75% (*p*=0.003) and the post-tumor board cohort had a better OS and a lower mortality risk (HR: 0.48) (14). In Germany, the guidelines of the oncologic societies and the National Cancer Plan as well force the implementation of MDTB as prerequisite standard of oncologic treatment to become a certified center. In our certified center’s weekly head & neck MDTB, the presence of at least two head neck surgeons, two maxillofacial surgeons, one radiologist, one pathologist, one radio-oncologist, and one oncologist is obligatory; further disciplines participate if necessary.

Pre-therapeutic presentation of LAOSCC cases in the MDTB and the standardized diagnostic workup and patient-centered decision making for surgery and neck dissection followed by risk-factor adapted adjuvant therapy improves LC and LRC in LAOSCC as demonstrated here in our retrospective analysis of 316 cases. Related to improved LC and LRC in cohort 2, there was a trend to improved outcome. Despite significantly improved LC and LRC, the DC appeared to be reduced (*p*=0.0033). A reduced DC/DMFS was also reported for neck squamous cell carcinoma of unknown primary tumors (8). Impaired DC in our sample was found to be significantly associated with N3 category (*p*=2.97∙10^-4^), localization in the tongue (C02; *p*=0.049), ECE+ (*p*=3.16∙10^-5^), and history of tobacco smoking (≤40 vs. >40 pack years, *p*=0.044). According to NCCN guidelines, unresectable disease demands CRT, and resectable N3 and/or ECE+ demand PORCT. Such LAOSCC patients received either 100 mg/m^2^ cisplatin in 3 cycles (days 1, 22, and 43) as recommended or fractionated in five doses of 20 mg/m^2^ days 1-5, 22-26, and 43-47 (7, 10). A tendency for loss of DC was found for cisplatin-based CRT or PORCT (*p*=0.059). The associations of reduced DC with high level smoking, localization of the OSCC in the tongue, presence of ECE+, T4 and N3 categories, and the consequent cisplatin-use are even more relevant as the loss of DC exerts the strongest impact on TSS and OS (**Figure 3**).

The particular reasons for the increased number of distant metastasis diagnosed in cohort 2 are currently unclear. One possible explanation is the increased use of more sensitive diagnostic tools (15). Our standardized workup and the staging procedure before the presentation of the case in the MDTB consists of at least a CT-scan of head and neck and the chest, either ultrasound or CT-scan of the abdomen, and a bone scan in all advanced stages (≥T3 or N+). Since 2006, a PET-CT system is available at our center, and [^18^F]-FDG-PET-CT scans are used for pre-therapeutic imaging of all cases presenting with bulky disease (≥T4a or N3), suspected residual disease after completed therapy or relapse. This functional imaging may have led to an earlier detection of otherwise occult asymptomatic distant metastasis and therefore linked to earlier detection of (at this time) rather treatable M1 but caused formally an impaired DC. Indeed, 16.7% vs. 47.4% distant failures (DF) were detected *before* loss in LRC in cohort 1 vs. 2. This may suggest that the DC could be impaired but OS and TSS have, nevertheless, improved to some extent. Due to the missing negative impact on survival, diagnostic improvements and use of PET/CT imaging in particular may have led to detection of otherwise occult distant metastases and caused the observed loss in DC. Early detection of single or oligo metastases and their surgical removal or irradiation may have contributed to prolonged OS in cohort 2 despite higher frequent loss of DC in cohort 2.

There was a difference between the two cohorts regarding participation in randomized controlled trials (RCT). In cohort 1, only 2 patients were included in an RCT but 35 patients of cohort 2 (and these patients underwent additional imaging including PET-CT). The increased use of CT and PET-CT imaging in patients within RCT may also have contributed to M1 detection: 26 of 35 LAOSCC patients in RCT of cohort 2 had M1 and 20 of them had their M1 diagnosis simultaneous to the LRC event leading to recommendation for systemic treatment and enrollment in one of the first-line RCT.

Since 2006, more LAOSCC (UICC Stage III-IVB, TNM 2010 and 2017) diagnosed underwent a complex risk factor-adapted multimodal treatment with curative intent. Specifically, more radical procedures were performed in a curative intent in cohort 2 in patients who before 2006 were declared being in a palliative state. However, patients with a more advanced tumor category and nodal metastasis (N+) have a higher risk for distant metastasis (15, 16). The proportion of tracheostomy, percutaneous gastrostomy and applied neck dissection increased, and consequently both the detection of N+ neck and ECE+ increased significantly, too.

During the study period, the rate of neck dissections increased. This is probably a main contributor to improved LRC. Before 2006, 42.3% of all patients did not undergo neck dissection whilst after 2006 only 28.3% did not. The increase in the number of neck dissections and the increased number of resected nodes examined by the pathologist per case led to a higher frequency of removed disease-positive nodes in particular. This change is attributable to the adherence and better implementation of the guidelines linked to decision-making and quality control of results in the MDTB. The benefit of an elective neck dissection for survival was demonstrated in cN0 oral cancer in several studies (5, 8, 10, 17). The mean number of assessed lymph nodes (nodal yield) increased from 10.1 before 2006 to 26.9 after 2006, respectively, suggesting the quality of neck dissection improved during the study period. The literature strongly suggests that a higher nodal yield is associated with a better survival and loco-regional control in head and neck cancer even when all dissected lymph nodes are negative. The optimal threshold for nodal yield in cN0 OSCC seems to be between 16 and 18 (18–20). Furthermore, the number of assessed nodes correlates with a higher N classification due to detection of occult lymph node metastases and detection of ECE+ as recorded in this study.

Cisplatin-based chemo-radiotherapy showed a tendency to predict impaired DC/DMFS and was found to predict distant metastasis in the PS-matched subgroup. Patients with larger tumor stages at diagnosis will more often receive platinum-based chemotherapy than those with smaller ones (21). Indeed, due to higher frequency in higher T and N categories, and in particular more T4 tongue cancer compared to cohort 1 and aiming on preventing glossectomy, patients in cohort 2 more often were treated by cisplatin-based CRT or PORCT (**Table 2**). Cisplatin seems to be unable to delete peripheral (circulating or already tissue-infiltrating) tumor cells completely for preventing distant metastasis in a patient cohort with more advanced disease (cohort 2).

As high level of smoking history (pack years), alcohol consumption, T1/T2 vs. T3/T4, N0 vs. N+, N3 vs. other N categories, of the primary LAOSCC sub-localization as well as age and sex were identified as independent predictive factors (*Pi*) for loss of DC (reduced DMFS) and, therefore, were expected to be significantly higher in LAOSCC-P of cohort 2 in general and hence potential confounders, logistic regression and propensity-score matching (PS-matching) was used to identify patients with identical or most similar characteristics in cohort 1 and 2). Cox hazard regression indeed identified N3, cisplatin-based PORCT/CRT and belonging to cohort 2 as independent predictors for losing DC in these 70 PS-matched LAOSCC-P pairs. However, the role of possible confounders and additional risk factors (*e.g.* smoking) and unwanted side effects of adjuvant treatment probably also linked to loss of DC have to be further clarified. However, it is conceivable that cisplatin not only could be unable to completely delete peripheral (circulating or already tissue-infiltrating) tumor cells and prevent distant metastasis in a patient cohort with more advanced disease (as in our cohort 2). However, belonging to cohort 2 emerged as independent predictor for distant metastasis in the 70 PS-matched LAOSCC-P, and therefore a closer look at differences into treatment regimens appear to be warranted.

Cisplatin-based CRT or PORCT may have the potential to trigger resistance to cisplatin and distant metastasis. Besides earlier observations that incomplete per-protocol treatment or use of inadequately low cisplatin doses are accompanied by loss of sensitivity to cisplatin treatment, recent papers highlight at least two additional mechanisms potentially involved in resistance to cisplatin and increased frequency of distant metastasis after cisplatin treatment due to unwanted side effects occurring whenever cisplatin is given combined with dexamethasone. Pan and collaborators demonstrated cisplatin-mediated activation of the glucocorticoid receptor (GR). The cisplatin-GR complex translocates into the nucleus. This complex induces platinum resistance *via* activating expression of MAST1, a critical platinum resistance factor component (22). A recent study by Zhang et al. demonstrated in various mice models pro-metastatic effects of dexamethasone *via* a PI3K-SGK1-CTGF pathway (23). Ligation of the GR by dexamethasone activated the PI3K signaling pathway and upregulated serum glucocorticoid-inducible kinase 1 (SGK1) expression, and then increased the expression of connective tissue growth factor (CTGF) through Nedd4l-Smad2 (23). Moreover, dexamethasone-induced SGK1 upregulated CTGF induced the expression of integrins Itgα6 and Itgβ1, and either SGK1 inhibition or CTGF knockdown downregulated these integrin genes. Interestingly, dexamethasone did not impair the response of the primary tumor towards paclitaxel in their *in vivo* models (23). This is different to Pan et al. (22) who demonstrated a prometastatic effect of dexamethasone combined with cisplatin and rather reduced efficacy of cisplatin after dexamethasone treatment. However, dexamethasone in concentrations and dosing schemata used in clinical routine induced increased migration of tumor cells and enhanced metastasis into the lung (23). This prometastatic effect was independent of immunosuppressive ability of dexamethasone (23).

Aiming on reducing chemo-related side effects, dexamethasone and histamine-receptor blockade ± neurokinin inhibitors were increasingly used since their approval and are part of guideline-conform treatment of LAOSCC (10). We noticed a 10% increase in use of cisplatin-based CRT and PORCT in cohort 2 (**Table 2**). Moreover, also treatment protocols were modified and cisplatin-based PORCT used more often the more fractionated schema applying the total cisplatin dose in 3 cycles of five daily doses each of 20 mg/m^2^ (days 1-5, 22-26, and 43-47) since 2007 (cohort 2) accompanied by prolonged adjuvant dexamethasone administration. Looking at dexamethasone dosing only in LAOSCC-P during CRT or PORCT, cohort 2 patients received dexamethasone on more days (10.1 *versus* 8.5 days; *p*=0.01018) summing up to a higher mean total dose (119.6 *versus* 100.1 mg dexamethasone; *p*=0.02032). Just for comparison: The mean total dexamethasone doses per patients calculated for all patients independent of receiving chemo-radiotherapy or not would result in 27.7 (95% CI 18.4 - 36.9) mg dexamethasone in cohort 1 and 40.5 (95% CI 32.4 - 48.6) mg dexamethasone in cohort 2 (*p*=0.04503). The association of higher proportion of patients receiving dexamethasone (also in higher mean dexamethasone doses) and higher frequency of distant metastasis in cohort 2 requires an explanation.

In the light of the studies by Pan et al. (22) and Zhang et al. (23) and other recent papers dealing with unwanted side effects of dexamethasone pointing towards elevated distant metastasis, either a reduction of dexamethasone use or targeting the GR signaling pathway components appear to be desirable. After demonstrating dexamethasone-induced cisplatin-resistant tumor growth *in vivo* in patient-derived xenograft (PDX) mouse models of head and neck cancer, Pan et al. demonstrated that treatment with lestaurtinib, an inhibitor of MAST1, fully revived cisplatin sensitivity in the dexamethasone-treated (prior cisplatin-resistant group) and even further attenuated tumor growth compared to the group treated with cisplatin alone (22). On the other hand, targeting SGK1 with a small molecule (GSK650394) could inhibit dexamethasone-induced lung metastasis without affecting antitumor capacity, as demonstrated in a murine breast cancer model (23). Besides aiming on a reduction of dexamethasone use in solid cancers including HNSCC, research is needed respective to hormone receptor signaling and its impact on distant metastasis and blocking these pathways, *e.g.* by using inhibitors for MAST1 (22) or SGK1 (23).

Since the implementation of a standardized MDTB in 2007, the mean time to intervention (TTI) in our cohort extends from 23.2 days to 32.8 days, respectively. Even after exclusion of TTI as a significant factor for DC, OS, TSS and other outcome measures (*p*>0.5 in all Cox models), the impact of this delay on OS and DC in our dataset remains unclear. A negative correlation of extended TTI on OS has been published by several authors (24–26). However, the delay in treatment initiation in our trial was not that huge as in the cited studies (45-68 days). Despite being without significant impact on outcome in our study, we are now aiming on shortening the interval between diagnosis and treatment to not potentially compromise the gains form MDTBs ensuring standardized cancer care and improve decision-making.

In our analysis, the patient volume increased from 6.5 cases per year to 21.2 cases per year. The impact of hospital and surgeon volume on the outcome of head and neck cancer patients was demonstrated in several trials (27–29). We assume that the increase in patient number is due to the improved structural and process quality (selective referral theory (27–29)) which is among other things certainly also a result of the implementation of the MDTB.

The limitations of our study are the small sample size in particular in subgroups analyzed and its retrospective nature. Inherent to the design we have to deal with missing data that could have impact on our results. Attributable to treatment of the most LAOSCC cases in routine (“real-world setting”), comorbidity led in a minority of cases to deviations between treatment recommended by the MDTB and applied. The distribution in localization of the primary lesions within the oral cavity and the shift towards higher age and presentation with more advanced tumors (T4) and higher N+ numbers and increased frequency in ECE+ reported represents a bias with not completely clarified impact on outcome including reduced DC in elderly patients and those with more advanced disease. Moreover, we could only report about a correlation between increased frequency of distant metastasis and increased use of cisplatin-based CRT and PORCT and simultaneously increased utilization of dexamethasone along altered fractionation protocols. This, however, remains an unproven hypothesis as long as evidence is missing that prolonged use of dexamethasone before and during chemotherapy to reduce acute toxicity and unwanted cisplatin-related side effects indeed comes at the price of an increase in distant metastasis and reduced DC in LAOSCC. However, one of the strength of our study is its intent-to-treat character and the validation of findings in sensitivity analyses based on propensity-score matched cases, which revealed stability of effects observed in comparison of both total cohorts and subgroups.

## Conclusion

Despite standardized diagnostic procedures, decision-making in the MDTB considering clear indications and improved therapy algorithms leading to improved LC and LRC only a slightly improved TSS and OS was achieved. The increased frequency of distant failure in cohort 2 accompanies changes in patient characteristics. Altered characteristics include age at diagnosis and increased proportions of T4 and N2-3 categories. Consequently, the use of cisplatin, BSC or palliative treatment according to patient’s preference increased. The identification of distant metastases, however, predominantly relates to diagnostic procedures during follow-up including use of advanced imaging methods including CT, MRI or PET-CT utilized in cohort 2. As simultaneous to cisplatin-based CRT and PORCT increased use of dexamethasone may have partly contributed to impaired DC based on its ability to induce expression of MAST1 (22) and of CTGF *via* SGK1 (23) the targeting of these critical proteins with inhibitors like lestaurtinib and GSK650394, respectively, may help to eliminate the unwanted side effects of steroids and re-sensitize LAOSCC and distant metastases to cisplatin treatment.

## Data Availability Statement

The raw data supporting the conclusions of this article will be made available by the authors, without undue reservation.

## Ethics Statement

The studies involving human participants were reviewed and approved by Ethics Committee of the University Leipzig. The patients/participants provided their written informed consent to participate in this study.

## Author Contributions

Conceptualization, GW. Methodology, GW. Validation, GW and MP. Formal analysis, GW and MP. Investigation, GW and MP. Resources, GW, DH, TK, RK, TG, BL, AD, SW, and VZ. Data curation, GW and MP. Writing—original draft preparation, GW and MP. Writing— review and editing, all authors. Visualization, GW and MP. Supervision, GW, SW, AD, and VZ. Project administration, GW. Funding acquisition, GW and AD. All authors contributed to the article and approved the submitted version.

## Funding

The study was partly supported by the grants LIFE-006 B7 and LIFE-007 D9 of the Leipzig Research Center for Civilization Diseases (LIFE), University Leipzig. LIFE is funded by the European Union, the European Fund for Regional Development (EFRE), and the Free State of Saxony. The funding sources did not influence the design of the study, collection, interpretation and analysis of the data, the preparation of this report, or the decision to publish.

## Conflict of Interest

The authors declare that the research was conducted in the absence of any commercial or financial relationships that could be construed as a potential conflict of interest.

## Publisher’s Note

All claims expressed in this article are solely those of the authors and do not necessarily represent those of their affiliated organizations, or those of the publisher, the editors and the reviewers. Any product that may be evaluated in this article, or claim that may be made by its manufacturer, is not guaranteed or endorsed by the publisher.
